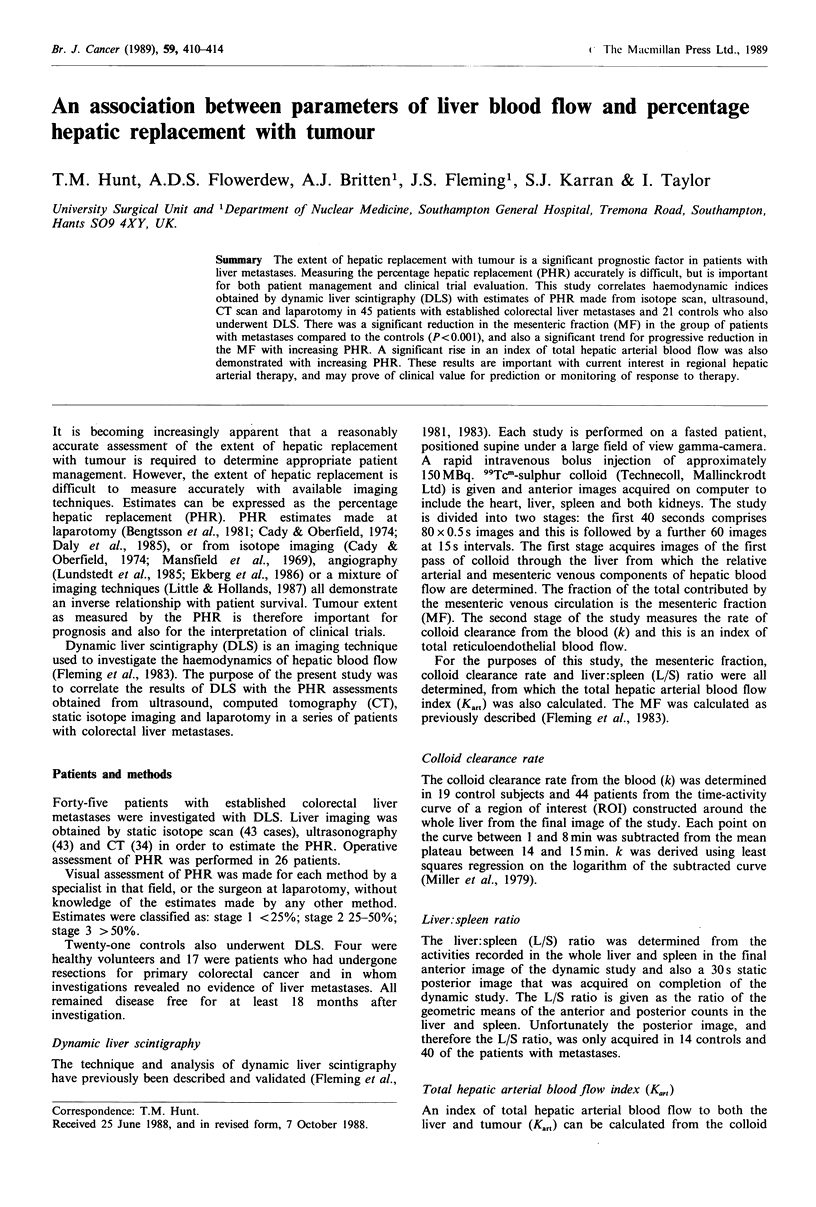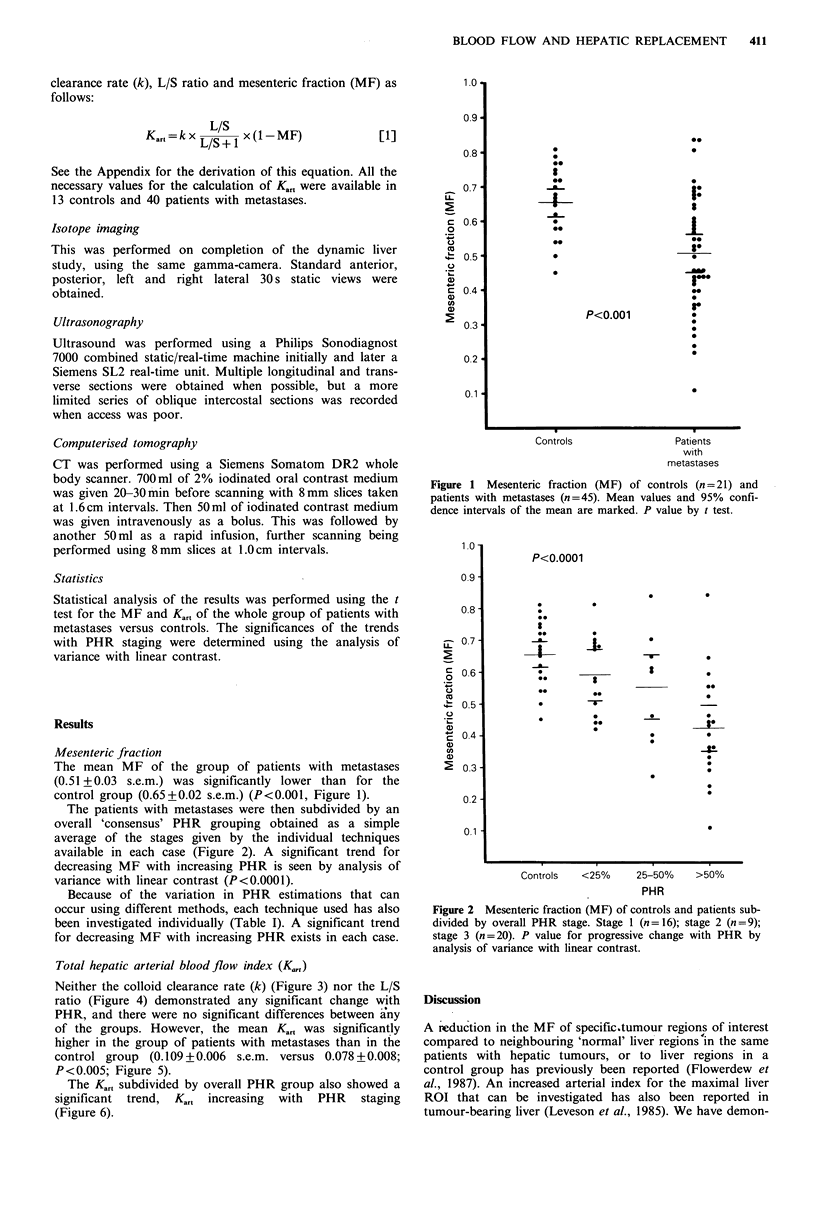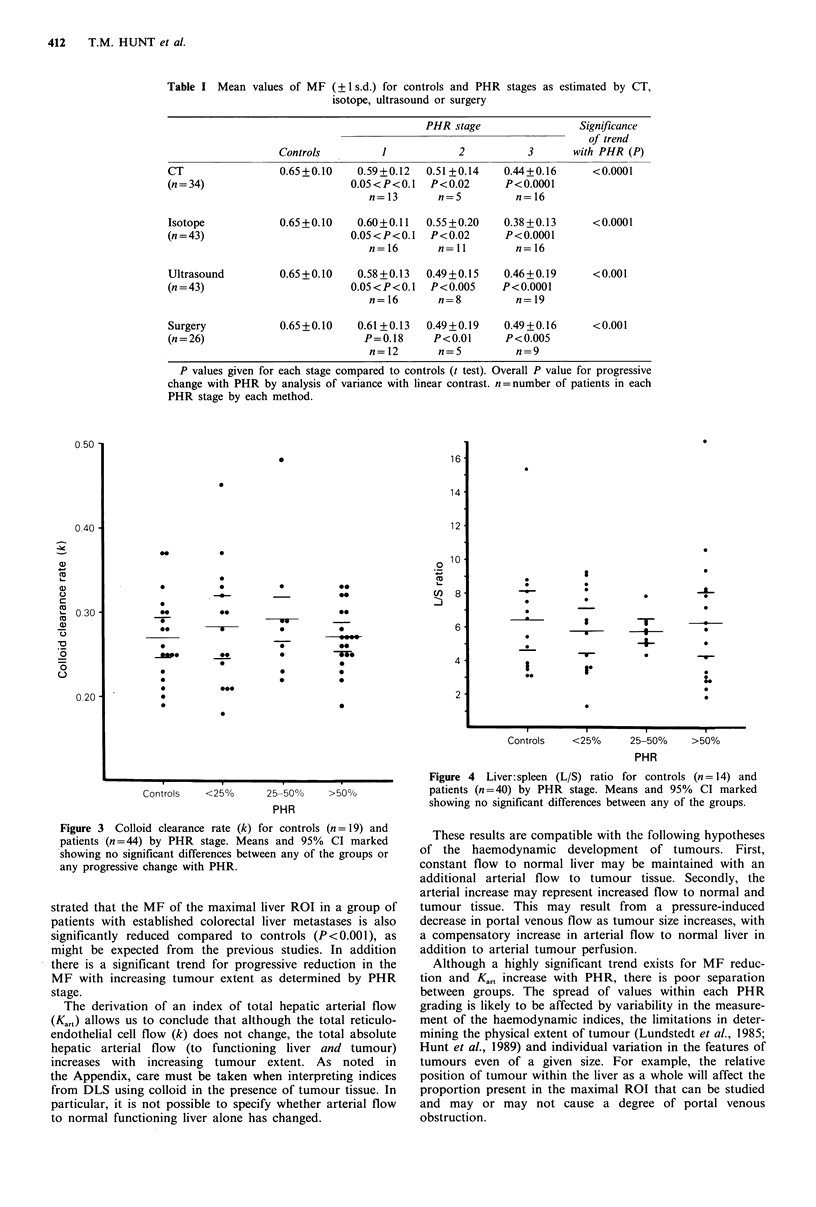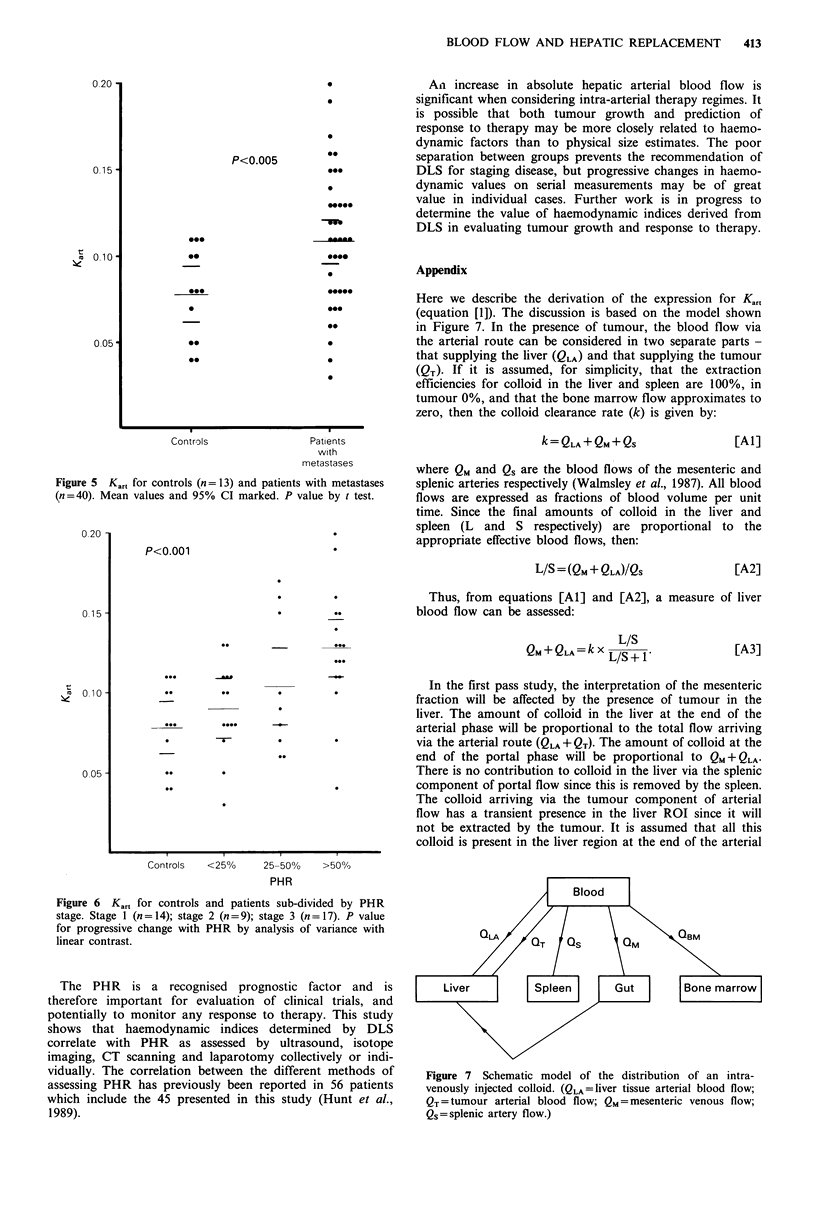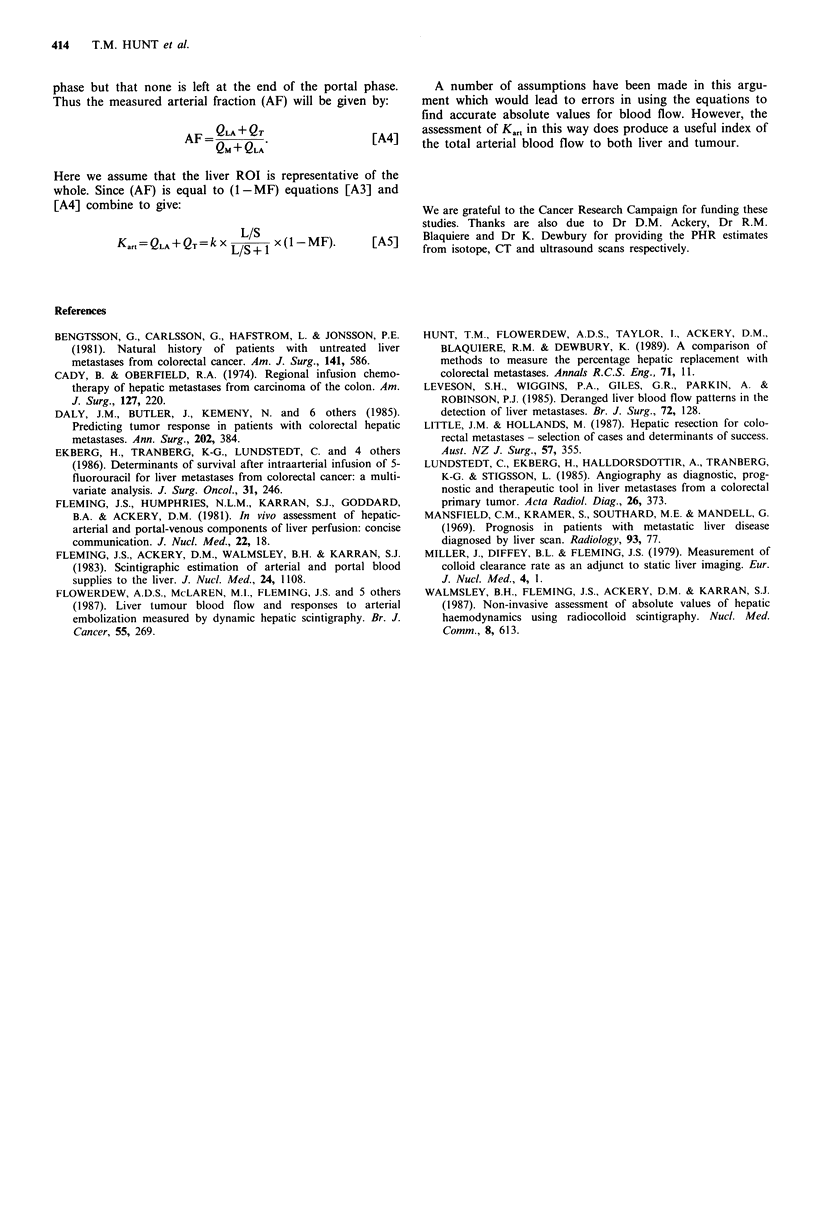# An association between parameters of liver blood flow and percentage hepatic replacement with tumour.

**DOI:** 10.1038/bjc.1989.82

**Published:** 1989-03

**Authors:** T. M. Hunt, A. D. Flowerdew, A. J. Britten, J. S. Fleming, S. J. Karran, I. Taylor

**Affiliations:** University Surgical Unit, Department of Nuclear Medicine, Southampton General Hospital, UK.

## Abstract

The extent of hepatic replacement with tumour is a significant prognostic factor in patients with liver metastases. Measuring the percentage hepatic replacement (PHR) accurately is difficult, but is important for both patient management and clinical trial evaluation. This study correlates haemodynamic indices obtained by dynamic liver scintigraphy (DLS) with estimates of PHR made from isotope scan, ultrasound, CT scan and laparotomy in 45 patients with established colorectal liver metastases and 21 controls who also underwent DLS. There was a significant reduction in the mesenteric fraction (MF) in the group of patients with metastases compared to the controls (P less than 0.001), and also a significant trend for progressive reduction in the MF with increasing PHR. A significant rise in an index of total hepatic arterial blood flow was also demonstrated with increasing PHR. These results are important with current interest in regional hepatic arterial therapy, and may prove of clinical value for prediction or monitoring of response to therapy.


Br. J. Cancer (1989), 59, 410–414 © The Macmillan Press Ltd., 1989

### An association between parameters of liver blood flow and percentage hepatic replacement with tumour

T.M. Hunt, A.D.S. Flowerdew, A.J. Britten[1], J.S. Fleming[1], S.J. Karran & I. Taylor

University Surgical Unit and [1]Department of Nuclear Medicine, Southampton General Hospital, Tremona Road, Southampton, Hants SO9 4XY, UK.

**Summary** The extent of hepatic replacement with tumour is a significant prognostic factor in patients with liver metastases. Measuring the percentage hepatic replacement (PHR) accurately is difficult, but is important for both patient management and clinical trial evaluation. This study correlates haemodynamic indices obtained by dynamic liver scintigraphy (DLS) with estimates of PHR made from isotope scan, ultrasound, CT scan and laparotomy in 45 patients with established colorectal liver metastases and 21 controls who also underwent DLS. There was a significant reduction in the mesenteric fraction (MF) in the group of patients with metastases compared to the controls ($P<0.001$), and also a significant trend for progressive reduction in the MF with increasing PHR. A significant rise in an index of total hepatic arterial blood flow was also demonstrated with increasing PHR. These results are important with current interest in regional hepatic arterial therapy, and may prove of clinical value for prediction or monitoring of response to therapy.

It is becoming increasingly apparent that a reasonably accurate assessment of the extent of hepatic replacement with tumour is required to determine appropriate patient management. However, the extent of hepatic replacement is difficult to measure accurately with available imaging techniques. Estimates can be expressed as the percentage hepatic replacement (PHR). PHR estimates made at laparotomy (Bengtsson *et al.*, 1981; Cady & Oberfield, 1974; Daly *et al.*, 1985), or from isotope imaging (Cady & Oberfield, 1974; Mansfield *et al.*, 1969), angiography (Lundstedt *et al.*, 1985; Ekberg *et al.*, 1986) or a mixture of imaging techniques (Little & Hollands, 1987) all demonstrate an inverse relationship with patient survival. Tumour extent as measured by the PHR is therefore important for prognosis and also for the interpretation of clinical trials.

Dynamic liver scintigraphy (DLS) is an imaging technique used to investigate the haemodynamics of hepatic blood flow (Fleming *et al.*, 1983). The purpose of the present study was to correlate the results of DLS with the PHR assessments obtained from ultrasound, computed tomography (CT), static isotope imaging and laparotomy in a series of patients with colorectal liver metastases.

### Patients and methods

Forty-five patients with established colorectal liver metastases were investigated with DLS. Liver imaging was obtained by static isotope scan (43 cases), ultrasonography (43) and CT (34) in order to estimate the PHR. Operative assessment of PHR was performed in 26 patients.

Visual assessment of PHR was made for each method by a specialist in that field, or the surgeon at laparotomy, without knowledge of the estimates made by any other method. Estimates were classified as: stage 1 <25%; stage 2 25–50%; stage 3 >50%.

Twenty-one controls also underwent DLS. Four were healthy volunteers and 17 were patients who had undergone resections for primary colorectal cancer and in whom investigations revealed no evidence of liver metastases. All remained disease free for at least 18 months after investigation.

### Dynamic liver scintigraphy

The technique and analysis of dynamic liver scintigraphy have previously been described and validated (Fleming *et al.*, 1981, 1983). Each study is performed on a fasted patient, positioned supine under a large field of view gamma-camera. A rapid intravenous bolus injection of approximately 150 MBq. $^{99}Tc^{m}$-sulphur colloid (Technecoll, Mallinckrodt Ltd) is given and anterior images acquired on computer to include the heart, liver, spleen and both kidneys. The study is divided into two stages: the first 40 seconds comprises $80\times0.5$ s images and this is followed by a further 60 images at 15 s intervals. The first stage acquires images of the first pass of colloid through the liver from which the relative arterial and mesenteric venous components of hepatic blood flow are determined. The fraction of the total contributed by the mesenteric venous circulation is the mesenteric fraction (MF). The second stage of the study measures the rate of colloid clearance from the blood ($k$) and this is an index of total reticuloendothelial blood flow.

For the purposes of this study, the mesenteric fraction, colloid clearance rate and liver:spleen (L/S) ratio were all determined, from which the total hepatic arterial blood flow index ($K_{art}$) was also calculated. The MF was calculated as previously described (Fleming *et al.*, 1983).

### Colloid clearance rate

The colloid clearance rate from the blood ($k$) was determined in 19 control subjects and 44 patients from the time-activity curve of a region of interest (ROI) constructed around the whole liver from the final image of the study. Each point on the curve between 1 and 8 min was subtracted from the mean plateau between 14 and 15 min. $k$ was derived using least squares regression on the logarithm of the subtracted curve (Miller *et al.*, 1979).

### Liver:spleen ratio

The liver:spleen (L/S) ratio was determined from the activities recorded in the whole liver and spleen in the final anterior image of the dynamic study and also a 30 s static posterior image that was acquired on completion of the dynamic study. The L/S ratio is given as the ratio of the geometric means of the anterior and posterior counts in the liver and spleen. Unfortunately the posterior image, and therefore the L/S ratio, was only acquired in 14 controls and 40 of the patients with metastases.

### Total hepatic arterial blood flow index ($K_{art}$)

An index of total hepatic arterial blood flow to both the liver and tumour ($K_{art}$) can be calculated from the colloid

Correspondence: T.M. Hunt.
Received 25 June 1988, and in revised form, 7 October 1988.

clearance rate ($k$), L/S ratio and mesenteric fraction (MF) as follows:

$$K_{art} = k \times \frac{L/S}{L/S+1} \times (1 - MF) \quad [1]$$

See the Appendix for the derivation of this equation. All the necessary values for the calculation of $K_{art}$ were available in 13 controls and 40 patients with metastases.

*Isotope imaging*

This was performed on completion of the dynamic liver study, using the same gamma-camera. Standard anterior, posterior, left and right lateral 30 s static views were obtained.

*Ultrasonography*

Ultrasound was performed using a Philips Sonodiagnost 7000 combined static/real-time machine initially and later a Siemens SL2 real-time unit. Multiple longitudinal and transverse sections were obtained when possible, but a more limited series of oblique intercostal sections was recorded when access was poor.

*Computerised tomography*

CT was performed using a Siemens Somatom DR2 whole body scanner. 700 ml of 2% iodinated oral contrast medium was given 20–30 min before scanning with 8 mm slices taken at 1.6 cm intervals. Then 50 ml of iodinated contrast medium was given intravenously as a bolus. This was followed by another 50 ml as a rapid infusion, further scanning being performed using 8 mm slices at 1.0 cm intervals.

*Statistics*

Statistical analysis of the results was performed using the $t$ test for the MF and $K_{art}$ of the whole group of patients with metastases versus controls. The significances of the trends with PHR staging were determined using the analysis of variance with linear contrast.

### Results

*Mesenteric fraction*

The mean MF of the group of patients with metastases ($0.51 \pm 0.03$ s.e.m.) was significantly lower than for the control group ($0.65 \pm 0.02$ s.e.m.) ($P < 0.001$, Figure 1).

The patients with metastases were then subdivided by an overall 'consensus' PHR grouping obtained as a simple average of the stages given by the individual techniques available in each case (Figure 2). A significant trend for decreasing MF with increasing PHR is seen by analysis of variance with linear contrast ($P < 0.0001$).

Because of the variation in PHR estimations that can occur using different methods, each technique used has also been investigated individually (Table I). A significant trend for decreasing MF with increasing PHR exists in each case.

*Total hepatic arterial blood flow index ($K_{art}$)*

Neither the colloid clearance rate ($k$) (Figure 3) nor the L/S ratio (Figure 4) demonstrated any significant change with PHR, and there were no significant differences between any of the groups. However, the mean $K_{art}$ was significantly higher in the group of patients with metastases than in the control group ($0.109 \pm 0.006$ s.e.m. versus $0.078 \pm 0.008$; $P < 0.005$; Figure 5).

The $K_{art}$ subdivided by overall PHR group also showed a significant trend, $K_{art}$ increasing with PHR staging (Figure 6).

**Figure 1** Mesenteric fraction (MF) of controls ($n=21$) and patients with metastases ($n=45$). Mean values and 95% confidence intervals of the mean are marked. $P$ value by $t$ test.

**Figure 2** Mesenteric fraction (MF) of controls and patients subdivided by overall PHR stage. Stage 1 ($n=16$); stage 2 ($n=9$); stage 3 ($n=20$). $P$ value for progressive change with PHR by analysis of variance with linear contrast.

### Discussion

A reduction in the MF of specific tumour regions of interest compared to neighbouring 'normal' liver regions in the same patients with hepatic tumours, or to liver regions in a control group has previously been reported (Flowerdew *et al.*, 1987). An increased arterial index for the maximal liver ROI that can be investigated has also been reported in tumour-bearing liver (Leveson *et al.*, 1985). We have demon-

**Table I** Mean values of MF (±1 s.d.) for controls and PHR stages as estimated by CT, isotope, ultrasound or surgery

| | Controls | PHR stage 1 | PHR stage 2 | PHR stage 3 | Significance of trend with PHR (P) |
|---|---|---|---|---|---|
| CT ($n=34$) | 0.65±0.10 | 0.59±0.12<br>$0.05<P<0.1$<br>$n=13$ | 0.51±0.14<br>$P<0.02$<br>$n=5$ | 0.44±0.16<br>$P<0.0001$<br>$n=16$ | <0.0001 |
| Isotope ($n=43$) | 0.65±0.10 | 0.60±0.11<br>$0.05<P<0.1$<br>$n=16$ | 0.55±0.20<br>$P<0.02$<br>$n=11$ | 0.38±0.13<br>$P<0.0001$<br>$n=16$ | <0.0001 |
| Ultrasound ($n=43$) | 0.65±0.10 | 0.58±0.13<br>$0.05<P<0.1$<br>$n=16$ | 0.49±0.15<br>$P<0.005$<br>$n=8$ | 0.46±0.19<br>$P<0.0001$<br>$n=19$ | <0.001 |
| Surgery ($n=26$) | 0.65±0.10 | 0.61±0.13<br>$P=0.18$<br>$n=12$ | 0.49±0.19<br>$P<0.01$<br>$n=5$ | 0.49±0.16<br>$P<0.005$<br>$n=9$ | <0.001 |

*P* values given for each stage compared to controls (*t* test). Overall *P* value for progressive change with PHR by analysis of variance with linear contrast. $n$ = number of patients in each PHR stage by each method.

**Figure 3** Colloid clearance rate ($k$) for controls ($n=19$) and patients ($n=44$) by PHR stage. Means and 95% CI marked showing no significant differences between any of the groups or any progressive change with PHR.

**Figure 4** Liver:spleen (L/S) ratio for controls ($n=14$) and patients ($n=40$) by PHR stage. Means and 95% CI marked showing no significant differences between any of the groups.

strated that the MF of the maximal liver ROI in a group of patients with established colorectal liver metastases is also significantly reduced compared to controls ($P<0.001$), as might be expected from the previous studies. In addition there is a significant trend for progressive reduction in the MF with increasing tumour extent as determined by PHR stage.

The derivation of an index of total hepatic arterial flow ($K_{art}$) allows us to conclude that although the total reticulo-endothelial cell flow ($k$) does not change, the total absolute hepatic arterial flow (to functioning liver *and* tumour) increases with increasing tumour extent. As noted in the Appendix, care must be taken when interpreting indices from DLS using colloid in the presence of tumour tissue. In particular, it is not possible to specify whether arterial flow to normal functioning liver alone has changed.

These results are compatible with the following hypotheses of the haemodynamic development of tumours. First, constant flow to normal liver may be maintained with an additional arterial flow to tumour tissue. Secondly, the arterial increase may represent increased flow to normal and tumour tissue. This may result from a pressure-induced decrease in portal venous flow as tumour size increases, with a compensatory increase in arterial flow to normal liver in addition to arterial tumour perfusion.

Although a highly significant trend exists for MF reduction and $K_{art}$ increase with PHR, there is poor separation between groups. The spread of values within each PHR grading is likely to be affected by variability in the measurement of the haemodynamic indices, the limitations in determining the physical extent of tumour (Lundstedt *et al.*, 1985; Hunt *et al.*, 1989) and individual variation in the features of tumours even of a given size. For example, the relative position of tumour within the liver as a whole will affect the proportion present in the maximal ROI that can be studied and may or may not cause a degree of portal venous obstruction.

**Figure 5** $K_{art}$ for controls ($n = 13$) and patients with metastases ($n = 40$). Mean values and 95% CI marked. *P* value by *t* test.

**Figure 6** $K_{art}$ for controls and patients sub-divided by PHR stage. Stage 1 ($n = 14$); stage 2 ($n = 9$); stage 3 ($n = 17$). *P* value for progressive change with PHR by analysis of variance with linear contrast.

The PHR is a recognised prognostic factor and is therefore important for evaluation of clinical trials, and potentially to monitor any response to therapy. This study shows that haemodynamic indices determined by DLS correlate with PHR as assessed by ultrasound, isotope imaging, CT scanning and laparotomy collectively or individually. The correlation between the different methods of assessing PHR has previously been reported in 56 patients which include the 45 presented in this study (Hunt *et al.*, 1989).

An increase in absolute hepatic arterial blood flow is significant when considering intra-arterial therapy regimes. It is possible that both tumour growth and prediction of response to therapy may be more closely related to haemodynamic factors than to physical size estimates. The poor separation between groups prevents the recommendation of DLS for staging disease, but progressive changes in haemodynamic values on serial measurements may be of great value in individual cases. Further work is in progress to determine the value of haemodynamic indices derived from DLS in evaluating tumour growth and response to therapy.

### Appendix

Here we describe the derivation of the expression for $K_{art}$ (equation [1]). The discussion is based on the model shown in Figure 7. In the presence of tumour, the blood flow via the arterial route can be considered in two separate parts – that supplying the liver ($Q_{LA}$) and that supplying the tumour ($Q_T$). If it is assumed, for simplicity, that the extraction efficiencies for colloid in the liver and spleen are 100%, in tumour 0%, and that the bone marrow flow approximates to zero, then the colloid clearance rate ($k$) is given by:

$$k = Q_{LA} + Q_M + Q_S \qquad [A1]$$

where $Q_M$ and $Q_S$ are the blood flows of the mesenteric and splenic arteries respectively (Walmsley *et al.*, 1987). All blood flows are expressed as fractions of blood volume per unit time. Since the final amounts of colloid in the liver and spleen (L and S respectively) are proportional to the appropriate effective blood flows, then:

$$L/S = (Q_M + Q_{LA})/Q_S \qquad [A2]$$

Thus, from equations [A1] and [A2], a measure of liver blood flow can be assessed:

$$Q_M + Q_{LA} = k \times \frac{L/S}{L/S + 1}. \qquad [A3]$$

In the first pass study, the interpretation of the mesenteric fraction will be affected by the presence of tumour in the liver. The amount of colloid in the liver at the end of the arterial phase will be proportional to the total flow arriving via the arterial route ($Q_{LA} + Q_T$). The amount of colloid at the end of the portal phase will be proportional to $Q_M + Q_{LA}$. There is no contribution to colloid in the liver via the splenic component of portal flow since this is removed by the spleen. The colloid arriving via the tumour component of arterial flow has a transient presence in the liver ROI since it will not be extracted by the tumour. It is assumed that all this colloid is present in the liver region at the end of the arterial

**Figure 7** Schematic model of the distribution of an intravenously injected colloid. ($Q_{LA}$ = liver tissue arterial blood flow; $Q_T$ = tumour arterial blood flow; $Q_M$ = mesenteric venous flow; $Q_S$ = splenic artery flow.)

phase but that none is left at the end of the portal phase. Thus the measured arterial fraction (AF) will be given by:

$$AF = \frac{Q_{LA} + Q_T}{Q_M + Q_{LA}}. \qquad [A4]$$

Here we assume that the liver ROI is representative of the whole. Since (AF) is equal to (1 − MF) equations [A3] and [A4] combine to give:

$$K_{art} = Q_{LA} + Q_T = k \times \frac{L/S}{L/S + 1} \times (1 - MF). \qquad [A5]$$

A number of assumptions have been made in this argument which would lead to errors in using the equations to find accurate absolute values for blood flow. However, the assessment of $K_{art}$ in this way does produce a useful index of the total arterial blood flow to both liver and tumour.

We are grateful to the Cancer Research Campaign for funding these studies. Thanks are also due to Dr D.M. Ackery, Dr R.M. Blaquiere and Dr K. Dewbury for providing the PHR estimates from isotope, CT and ultrasound scans respectively.

### References

BENGTSSON, G., CARLSSON, G., HAFSTROM, L. & JONSSON, P.E. (1981). Natural history of patients with untreated liver metastases from colorectal cancer. *Am. J. Surg.*, **141**, 586.

CADY, B. & OBERFIELD, R.A. (1974). Regional infusion chemotherapy of hepatic metastases from carcinoma of the colon. *Am. J. Surg.*, **127**, 220.

DALY, J.M., BUTLER, J., KEMENY, N. and 6 others (1985). Predicting tumor response in patients with colorectal hepatic metastases. *Ann. Surg.*, **202**, 384.

EKBERG, H., TRANBERG, K-G., LUNDSTEDT, C. and 4 others (1986). Determinants of survival after intraarterial infusion of 5-fluorouracil for liver metastases from colorectal cancer: a multivariate analysis. *J. Surg. Oncol.*, **31**, 246.

FLEMING, J.S., HUMPHRIES, N.L.M., KARRAN, S.J., GODDARD, B.A. & ACKERY, D.M. (1981). *In vivo* assessment of hepatic-arterial and portal-venous components of liver perfusion: concise communication. *J. Nucl. Med.*, **22**, 18.

FLEMING, J.S., ACKERY, D.M., WALMSLEY, B.H. & KARRAN, S.J. (1983). Scintigraphic estimation of arterial and portal blood supplies to the liver. *J. Nucl. Med.*, **24**, 1108.

FLOWERDEW, A.D.S., McLAREN, M.I., FLEMING, J.S. and 5 others (1987). Liver tumour blood flow and responses to arterial embolization measured by dynamic hepatic scintigraphy. *Br. J. Cancer*, **55**, 269.

HUNT, T.M., FLOWERDEW, A.D.S., TAYLOR, I., ACKERY, D.M., BLAQUIERE, R.M. & DEWBURY, K. (1989). A comparison of methods to measure the percentage hepatic replacement with colorectal metastases. *Annals R.C.S. Eng.*, **71**, 11.

LEVESON, S.H., WIGGINS, P.A., GILES, G.R., PARKIN, A. & ROBINSON, P.J. (1985). Deranged liver blood flow patterns in the detection of liver metastases. *Br. J. Surg.*, **72**, 128.

LITTLE, J.M. & HOLLANDS, M. (1987). Hepatic resection for colorectal metastases – selection of cases and determinants of success. *Aust. NZ J. Surg.*, **57**, 355.

LUNDSTEDT, C., EKBERG, H., HALLDORSDOTTIR, A., TRANBERG, K-G. & STIGSSON, L. (1985). Angiography as diagnostic, prognostic and therapeutic tool in liver metastases from a colorectal primary tumor. *Acta Radiol. Diag.*, **26**, 373.

MANSFIELD, C.M., KRAMER, S., SOUTHARD, M.E. & MANDELL, G. (1969). Prognosis in patients with metastatic liver disease diagnosed by liver scan. *Radiology*, **93**, 77.

MILLER, J., DIFFEY, B.L. & FLEMING, J.S. (1979). Measurement of colloid clearance rate as an adjunct to static liver imaging. *Eur. J. Nucl. Med.*, **4**, 1.

WALMSLEY, B.H., FLEMING, J.S., ACKERY, D.M. & KARRAN, S.J. (1987). Non-invasive assessment of absolute values of hepatic haemodynamics using radiocolloid scintigraphy. *Nucl. Med. Comm.*, **8**, 613.